# The Applications of 3D-Printing Technology in Prosthodontics: A Review of the Current Literature

**DOI:** 10.7759/cureus.68501

**Published:** 2024-09-03

**Authors:** Mohammed H Alyami

**Affiliations:** 1 Prosthetic Dental Science, Najran University, Najran, SAU

**Keywords:** dental implants, prosthodontics, dental prosthesis, additive manufacturing, 3d printing

## Abstract

Prosthodontics has become increasingly popular because of its cosmetic attractiveness. 3D printing has revolutionized prosthodontics, enabling the creation of high-quality dental prostheses. It creates detailed restorations, such as crowns, bridges, implant-supported frameworks, surgical templates, dentures, and orthodontic models. In addition, it saves production time but faces challenges such as elevated expenses and the requirement for innovative materials and technologies. This review gives insights into the uses of 3D printing in prosthodontics, presenting how it has significantly changed clinical practices. This article discusses different materials and techniques. Additionally, it showcases the capacity of 3D printing to improve prosthodontic practice and proposes prospects for future investigation.

## Introduction and background

Prosthodontics and 3D printing in dentistry

Prosthodontics is a vital branch of dentistry focused on enhancing oral function, smile esthetics, and nutritional intake by replacing missing teeth with prostheses that mimic natural teeth. The increasing need for prosthodontic services is impacted by factors other than just a population that is getting older. Insufficient dental health awareness and a focus on aesthetics can also result in tooth loss. These factors emphasize the importance of advanced prosthodontic treatments that improve restorations' durability while preserving a harmonious connection between the prosthesis and the periodontium [1].

3D printing is a rapidly advanced technology that produces physical objects by adding layers based on digital designs constructed by digital software such as computer-aided design (CAD) [2]. This software generates restorations from scratch by storing volumetric data from intraoral scans. This technology is primarily used by professional dentists and technicians, offering a wide range of options and enhancing decision-making [3]. It has replaced traditional methods of fabricating dental prostheses, which were often time-consuming and required manual skills. 3D printing has revolutionized the creation of high-quality dental prostheses by allowing for precise customization and replication of complex anatomical structures. This technology enables the accurate modelling of intricate dental injuries and defects, which in turn facilitates more effective and tailored solutions in maxillofacial surgeries. By producing prostheses that are uniquely suited to the patient's anatomy, 3D printing has significantly advanced the field of maxillofacial surgery, improving surgical outcomes and patient satisfaction [4]. The accuracy of 3D printers in laboratories and clinics depends on several factors, including the scanner, scanning technique, operator expertise, and printer type [5].

Search strategy

A comprehensive search was conducted across several databases, including PubMed, Google Scholar, and Scopus. The search strategy employed the following keywords: ("3D printing" AND "prosthodontics" AND "CAD" AND "digital impression"). This review aims to trace the evolution of 3D-printing technology in prosthodontic treatments, highlighting its advancements and identifying areas requiring further research and development. While conventional and digital impressions were initially examined in detail, the focus has been streamlined to emphasize the application of 3D-printing technologies across various prosthodontic fields, such as removable, fixed, implant, and maxillofacial prosthodontics.

## Review

Historical overview

The evolution of dental prostheses has been remarkable, transitioning from rudimentary materials, such as wood, ivory, and animal teeth, to advanced technological solutions. During the Renaissance, the complexity of prosthetic devices increased notably, with metals becoming a key material due to their durability and workability. Early methods of prosthesis construction involved traditional materials such as plaster and alginate. However, these methods were often cumbersome and could compromise the accuracy of the final prosthetic outcomes [6,7]. The introduction of dental radiography represented a pivotal advancement, allowing for detailed evaluation of bone structures. This technological leap enhanced the planning process for implant-supported prostheses and improved the detection of pathologies that could impact prosthetic success [8].

In the last few years, 3D printers replaced traditional impressions and offered precise 3D digital models. For example, CAD/CAM modalities reduce errors and improve fit. At the same time, finite element analysis provides insights into the biomechanical behaviour of restorations, aiding in virtual stress testing for durability and longevity [9]. Modern digital smile design and mock-up techniques enable patients to preview esthetic outcomes before treatment, improving communication and aligning expectations [10]. Implant-supported design and surgical techniques have achieved high success rates and patient satisfaction. Novel materials such as high-strength ceramics and hybrid composites are superior for prosthetic restorations [11].

Conventional impressions and dental impressions facilitate the creation of precise models of oral structures that help in processing prosthesis fabrication and orthodontics [6]. Traditional impressions have been used in prosthodontics and dental implantology for years. Historically, materials such as beeswax and gypsum were used to create basic models of teeth and jaws. Conventional impressions typically use alginate, an elementary material derived from seaweed, for preliminary impressions due to its ease of use and cost-effectiveness. Additionally, it has limitations such as short working time and susceptibility to deformation [12].

**Figure 1:**
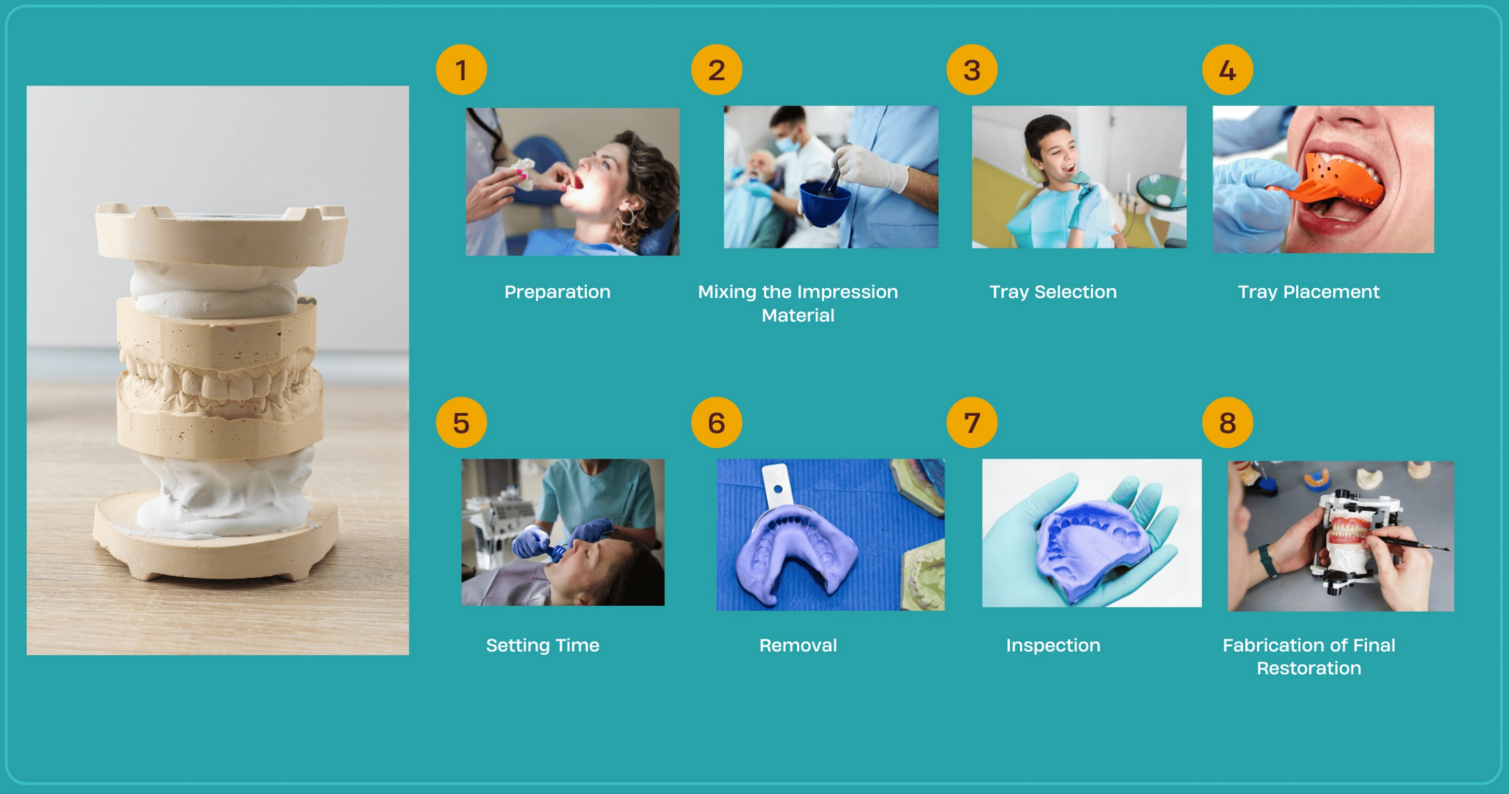
Traditional impression steps. We used the Canva application to create the figures. The included figures were published under a Creative Commons license.

The process involves preparing the patient, collecting and mixing impression materials, loading them into a tray, positioning the tray in the oral cavity, allowing the material to set, and capturing the dental structures. Once the impression material is removed and examined for accuracy, a stone or plaster cast is poured into the impression to create a detailed and precise physical model of the oral structures, as shown in Figure 1 [13]. While traditional impressions offer familiarity, affordability, and suitability for various procedures, they are prone to errors such as air bubble entrapment, material tearing, and distortions during removal, often causing patient discomfort [14].

Recently, digital impressions have gained popularity by offering several advantages over traditional methods. Digital scanners offer exceptional accuracy for capturing detailed 3D versions of oral anatomy with minimal discomfort and reduced waiting time. Digital scans can be stored electronically, which aids in simplifying their access, sharing, and integration with computer-assisted design and manufacturing systems for the distinct fabrication of dental prosthetics and planning for implants [14].

Methods and applications of 3D printing

3D printing encompasses various techniques, including binder jetting, material extrusion, powder bed fusion, stereolithography (SLA), digital light processing (DLP), and selective laser sintering (SLS). Each method has distinct characteristics and applications, contributing to the diverse capabilities of 3D printing in prosthodontics and other fields [15,16]. The cost of implementing 3D printing in dental practices can be a significant barrier [17,18]. Nevertheless, pioneers in the industry have respected the benefits of 3D printing [19]. While the benefits of 3D application for printing dental prosthetics are high, its widespread is limited due to challenges such as insufficient knowledge and training for staff [2].

Evolution of 3D-printing technology

Important landmarks have marked the evolution of 3D-printing technology. Initially, it was developed in the 1970s and gained support in the 1980s by Charles Hull. Since then, 3D printing has advanced through many types such as SLA, SLS, and fused deposition modeling (FDM) [20]. Current developments in 3D printing include various advancements that have major implications for multiple industries. These advancements involve new materials, such as waste materials and recycled sand, to improve sustainability [21]. Novel research has shown that 3D printing resulted in better results for prosthetic fit, aesthetic appeal, and patient satisfaction [22].

A case study assessing the functional performance and patient satisfaction of 3D-printed prostheses found that 3D-printed prostheses, although demonstrating better functional performance and improved gross manual dexterity as measured by the Box and Block test, received lower overall patient satisfaction compared to the standard prosthesis. The 3D-printed prosthesis also enhanced bimanual coordination, but concerns about its durability and effectiveness led to a preference for the standard-hook device, which scored higher in patient satisfaction surveys [23]. Patient satisfaction is influenced by challenges such as high production costs, slow printing rates, limited size options, and reduced material strength [24]. Additionally, the sustainability of 3D printing in this context is limited by the reliance on plastic materials. The medical industry also faces significant challenges in ensuring the safety and effectiveness of 3D-printed prostheses, necessitating rigorous testing and validation processes [25]. Despite these challenges, 3D printing has contributed to innovative approaches in treatment, training, and research. Moreover, the integration of 3D printing with AI holds great potential for advancing predictive dental care through AI-driven analysis [26].

3D-printing technologies

**Figure 2:**
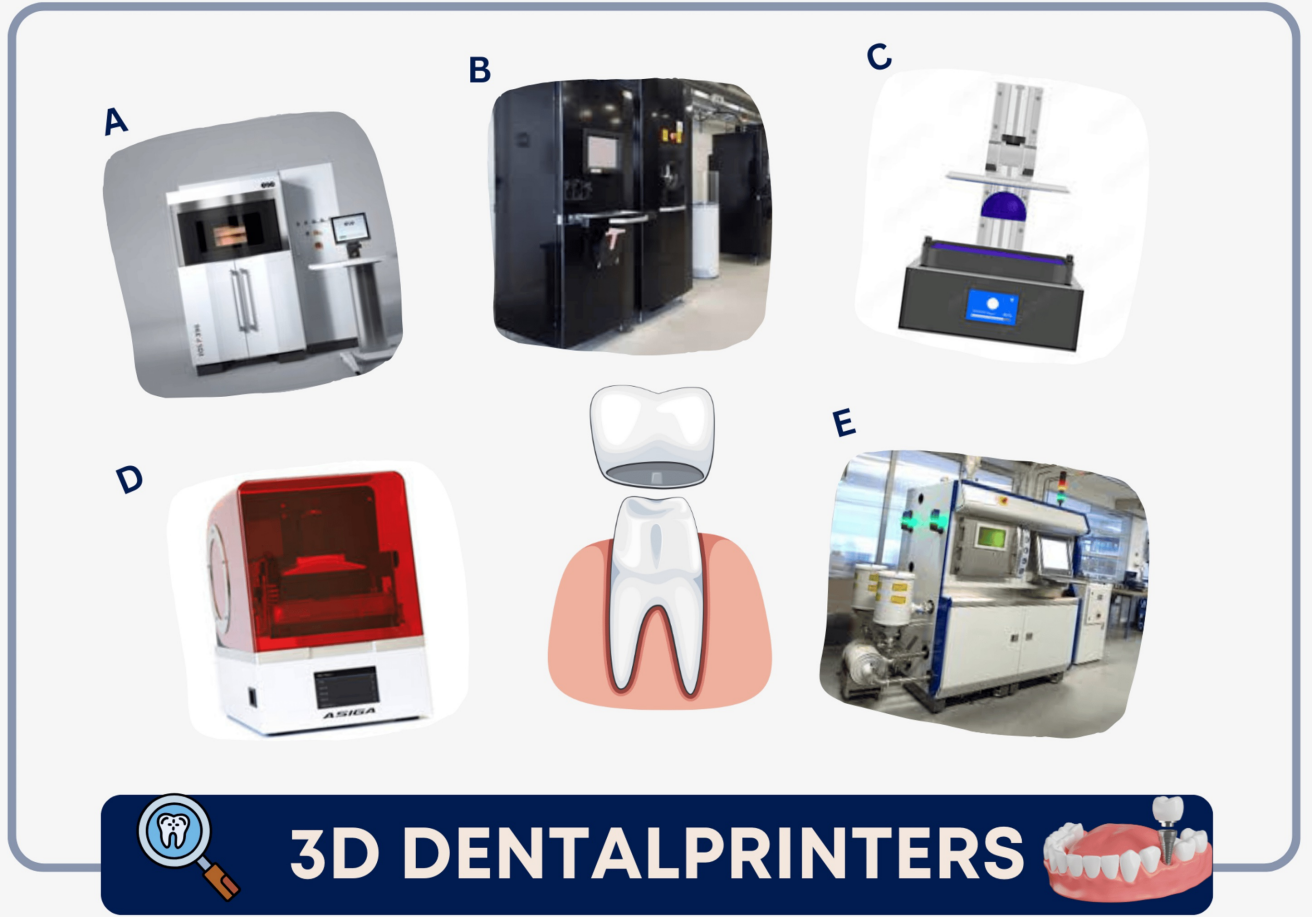
3D-printing technologies. A refers to selective laser sintering printers, B refers to electron beam melting 3D printers, C refers to stereolithography printers, D refers to fused deposition modeling printers, and E refers to direct metal laser sintering printers. We used the Canva application to create the figures. The included figures were published under a Creative Commons license.

3D-printing methods offer numerous advantages across various areas of dental practice. Significant progress is being observed in orthodontics, endodontics, prosthodontics, and periodontics due to the application of 3D printing technology. Each technique has a specific printer, as shown in Figure 2 [27]. To sum up, Table 1 covers various 3D-printing techniques and their main features in additive manufacturing.

Prosthodontics relies heavily on VAT photo-polymerization (VP) technologies, such as SLA and DLP, to create prosthesis bases with superior properties to traditional ones. Additional research should focus on improving mechanical properties and biocompatibility, also paying close attention to design and printing orientation, with in-vivo testing required for clinical assessment. Legal and regulatory standards must be followed when using them [28].

3D-printing methods

SLA

SLA is a widely used 3D-printing method in prosthodontics, particularly for creating custom dental prostheses such as crowns and bridges. This technology has progressively replaced conventional fabrication methods due to its capacity to produce complex structures with intricate geometries. Furthermore, SLA finds application in orthodontics for the manufacturing of aligners and other orthodontic appliances [29].

DLP

DLP is a rapid printing method. it is used for resin designs. Similar to SLA, DLP uses a light source to cure layers at once, which aids in speeding up the process. It is widely used in prosthodontics for dental restorations and orthodontic models. Although DLP technology enhances the mechanical and antibacterial properties of polymethyl methacrylate (PMMA) composite resin for dental applications, it is restricted to photopolymers that emit odors, which can be an issue in office environments [30].

FDM

FDM has gained significant traction in prosthodontics due to its accessibility, cost-effectiveness, and versatility. This technology excels in creating complex diagnostic models, surgical guides, and orthodontic appliances. For instance, FDM-produced surgical guides have demonstrated improved accuracy and efficiency in implant placement procedures, leading to reduced surgical time and increased patient satisfaction. Additionally, FDM-fabricated orthodontic models provide precise representations of dental arches, aiding in treatment planning and appliance design. Rapid prototyping (RP) capabilities offered by FDM facilitate the iterative design and evaluation of dental devices, accelerating the development process [31,32].

SLS

SLS is applied for dental frameworks and bases. It uses a laser to fuse powdered material layers to create 3D objects. This technology employs a laser to selectively fuse powdered material, layer by layer, to create precise 3D objects. This process results in highly detailed and accurate models with natural contours, making it particularly well-suited for complex dental conditions. However, it has defects such as possible inhalation hazards from particles, messy disposal of extra powder, and slower production speed compared to certain 3D-printing modalities [33].

Electron Beam Melting (EBM)

EBM is an advanced additive manufacturing technique that uses an electron beam to melt metal powder, creating high-quality 3D structures. In the dental and maxillofacial fields, it is utilized for a range of applications, including dental implants, customized implant abutments, precise orthodontic appliances, and accurate surgical templates. Additionally, EBM is increasingly used to fabricate dental restorations, as well as specialized maxillofacial implants for facial reconstruction [34]. A case study successfully demonstrated the reconstruction of a mandibular defect using a patient-specific prosthesis fabricated via EBM. This innovative approach resulted in significantly reduced surgical time, excellent aesthetic outcomes, and high patient satisfaction, as measured by the EORTC QLQ-C30 and H&N35 quality-of-life questionnaires. The prosthesis incorporated a unique design combining porous regions for bone ingrowth and solid load-bearing areas to optimize both biological and mechanical functions. Over a nine-month follow-up, the implant exhibited excellent integration without complications [35].

Direct Metal Laser Sintering (DMLS)

DMLS uses powerful lasers to melt and combine metal powder particles. It makes durable restorations from materials such as cobalt-chromium and titanium metals. Although this technique provides better fit, and quality than traditional methods, its high cost is an important consideration in dental manufacturing [36].

Material Jetting

Material jetting is an advanced 3D-printing technique that layers liquid materials to create dental restorations. In prosthodontics, it offers advantages such as high precision and biocompatibility, allowing for the production of restorations. Furthermore, material jetting enables RP, speeding up design and modification processes. Frequently used materials include photocurable resins for their accuracy, ceramics for their aesthetic appeal and biocompatibility, and metal alloys for their durability in implants and frameworks. This technology significantly enhances the quality and efficiency of dental restoration fabrication [27].

**Table 1:** Summary table of 3D-printing technologies.

3D-Printing Technology	Main Materials	Characteristics	Specific Applications	References
Fused Deposition Modeling (FDM)	Thermoplastics (e.g., PLA, ABS, PETG)	Simple and cost-effective process. Good mechanical strength. Layer adhesion may affect surface finish and strength. Widely used for rapid prototyping and functional parts.	Orthodontic models, surgical guides, dental aligners	Dave et al., 2021; Kim et al., 2020 [31,32]
Stereolithography (SLA)	Photopolymer resins	High-resolution and surface quality. Suitable for intricate designs. Requires post-curing for optimal mechanical properties. Use UV light to cure resin layer by layer.	Dental crowns, bridges, and implant guides	Zeng et al., 2023 [29]
Selective Laser Sintering (SLS)	Powdered thermoplastic (Nylon, PA12, TPU)	No need for support structures. Good mechanical properties. Suitable for functional prototypes and end-use parts. Laser fuses powder particles to create solid objects.	Dental frameworks, orthodontic devices	Fina et al., 2017 [33]
Digital Light Processing (DLP)	Photopolymer resins	Fast printing speed. High resolution. Limited to smaller build volumes compared to SLA.	High-precision dental models, prosthodontic appliances	Muhindo et al., 2023 [30]
Binder Jetting	Sand, metal powders	High-speed and cost-effective for large parts. Suitable for metal and sand-casting molds. Post-processing is required for part strength. Ideal for large-scale production and sand-casting molds.	Dental models, metal frameworks	Kasihani et al., 2023; Sood et al., 2022 [15,16]
Electron Beam Melting (EBM)	Metal powders (e.g., titanium, aluminium)	High precision and surface finish. Suitable for complex metal parts. Requires vacuum environment for processing.	Dental implants, custom abutments	Suska et al., 2016 [35]
Direct Metal Laser Sintering (DMLS)	Metal powders (e.g., stainless steel, titanium)	High strength and density. Suitable for complex metal parts with tight tolerances. Expensive compared to other methods.	Metal prosthetics, dental implants	Sharma et al., 2021 [36]
Material Jetting	Photopolymers, wax	High resolution and accuracy. Multiple materials and colours can be used simultaneously. Higher cost and slower compared to other methods.	Detailed dental models, aesthetic restorations	Jeong et al., 2023 [27]

Process of 3D printing in prosthodontics

The process of 3D printing in prosthodontics involves capturing detailed digital scans of a patient's oral anatomy to create a precise 3D model. This virtual representation serves as the basis for designing and manufacturing various prosthetic components, such as crowns, dentures, and implant-supported restorations, through 3D-printing technologies [37,38].

Dental prosthesis fabrication with 3D printing has revealed promoting outcomes, with materials such as cobalt-chromium. However, additional research is required for materials such as zirconia. Predictably, 3D printing in dentistry will grow, providing many benefits [39].

Steps of digital printing

Digital Design and CAD Modeling

The manufacturing process of 3D-printing technology begins with digital design and CAD modelling [40]. Digital technology is used in all stages of creating oral prostheses, incorporating preoperative simulation analysis tools such as digital line-plane design (DLD) and digital smile design (DSD). According to maxillofacial prosthodontics, a similar process aids in capturing defect information and using specific software for creating maxillofacial prostheses. Moreover, CAD/CAM technology has had a major influence on orthodontics by allowing for virtual treatment planning, assisting with diagnosis, clear aligners, and appliances and speeding up orthodontic treatment. Integrating digital design with CAD modelling improves accuracy, productivity, and results for different dental prosthetics [27].

For example, a recent study compared the effectiveness of two removable die systems, namely, the Pindex and Di-Lok tray, in creating working casts from an implant master die with dental implant analogues. Each system produced 10 casts, which underwent thorough evaluation. The findings revealed significant differences in horizontal and vertical linear distances compared to intra-oral scans. Ultimately, the study concluded that intra-oral scans within a complete digital workflow provided distinct advantages over partial digital workflows employing extra-oral scans with both the Pindex and Di-Lok tray systems [41].

Material Selection and Preparation

Choosing and preparing materials for prosthodontics is essential for reaching the best results in digital printing. Specific materials such as polymethyl methacrylate (PMMA), metal powders, and polymeric systems are selected. PMMA grades processed by DLP 3D printers are evaluated based on mechanical, tribological, and economic factors, thereby enhancing efficiency in prosthetic treatments [42,43]. The metal powders used for 3D-printing dental prosthetics contain elements such as Cr, Mo, and Ag, which meet performance standards and have antibacterial properties. Examination of polymeric systems for temporary dental prostheses indicates that mechanical properties are significantly influenced by composition, fabrication mode, and thermal cycling, with bis-acryl systems demonstrating superior flexural strength [6,43].

Printing and Layer-by-Layer Fabrication

Printing and layer-by-layer fabrication procedures aid in developing prosthodontics by enabling the creation of complex 3D structures. RP methods such as SLA, SLA, and fused deposition modelling have significantly impacted various dental fields, including implantology and maxillofacial prosthodontics. These methods reduce human dependence, overcoming conventional limitations and offering detailed, effective fabrication of dental prostheses [38]. Investigations show that the thickness of the printing layer greatly affects the accuracy of temporary fixed and partial dentures created through additive manufacturing. Conversely, subtractive manufacturing methods generally exhibit greater accuracy regardless of layer thickness [24,27].

Post-Processing Finishes

Post-processing is essential for maintaining dimensional stability, material properties, and surface integrity of dental prostheses. Techniques such as post-curing with photopolymer resin can improve the mechanical properties of printed denture bases [44]. In addition, thermomechanical techniques can improve metal-ceramic crowns and prosthetic bridges by reducing porosity and enhancing material properties. Proper post-curing methods reduce deformation and improve the accuracy of 3D-printed prostheses [45].

Quality Control

Quality control is fundamental in fabricating 3D-printed prostheses to confirm that they meet specified requirements and industry standards. This applies different inspection procedures, including dimensional measurement, surface roughness analysis, and non-destructive testing. Dimensional measurement provides that the prostheses coordinate to the exact size and shape specifications. Surface roughness analysis considers the consistency and smoothness of the surface, which affects both functionality and comfort. Non-destructive testing techniques, such as X-ray or ultrasound, evaluate the internal structure and integrity of the prostheses deprived of causing damage [46].

Printing Materials for Dental Prostheses

Dental prostheses need long-lasting aesthetic materials for printing. Resin-based materials such as photopolymer and biocompatible resins are commonly used for dental models due to their benefits [47]. Polylactic acid is used for fabricating temporary crowns with fused deposition modelling printers [6,48]. Polyesters and polyamides are used in FDM printers to provide biodegradability and resilience, making them suitable for aligners [49].

Polymers are essential in impressions, commonly using polyvinyl siloxanes and polyethers for good application of oral structures [50]. Styrene polymers such as polystyrene and acrylonitrile butadiene styrene are usually used in printing temporary crowns and orthodontic models due to their good mechanical properties and ease of printing [51]. Methacrylates and urethane acrylates are favoured in photopolymer-based technologies such as SLA and DLP, which show excellent resolution and biocompatibility for permanent prosthodontics [52].

Materials such as hydroxyapatite, tricalcium phosphate, stem cells, collagen, and titanium alloys are applied in maxillofacial reconstructive surgery and implants [53]. Cobalt-chromium and titanium alloys are commonly chosen for crowns and bridges, whereas zirconia and lithium disilicate ceramics are preferred for veneers and bridges [54]. Ceramics are gaining popularity because of their attractive appearance, capacity to interact effectively with living things, and remarkable resistance to damage [55].

Composite materials, including polymers, ceramics, and metals, provide strength, and aesthetic flexibility. Options such as fibre-reinforced composites, nanocomposites, and hybrid materials make high-performed dental prostheses to meet specific patients' needs [56]. For instance, polyether ether ketone (PEEK) is used for dental implant frameworks [57,58].

We performed a comprehensive review of materials used in 3D printing and created a summary of our findings in Table 2.

**Table 2:** Comprehensive summarization of 3D-printing materials and their applications.

Material type	Characteristics	Clinical applications	Case study	Summary of the case study	References
Photopolymer Resins	High resolution, smooth surface finish	Crowns and bridges, temporary prostheses, orthodontic models and aligners	Evaluation of photopolymer resins for dental prosthetics fabricated via the stereolithography process at different polymerisation temperatures—Part I: Conversion rate and mechanical properties	A study conducted a power analysis to determine sample size and evaluated a photopolymer resin for interim dental prostheses. It examined density, viscosity, and mechanical properties at different polymerization temperatures using a stereolithography 3D printer. Results indicated that higher printing temperatures significantly improved double bond conversion (DBC) and mechanical properties. Specimens printed at 70°C showed optimal DBC, tensile stress, and strain. These findings suggest that increasing printing temperatures enhances the conversion rate and mechanical performance of printed parts, potentially prolonging the durability of dental prostheses. However, the study's limitations, including testing only one resin type and conducting in vitro assessments, underscore the need for further research with diverse materials and clinical validations.	Lee et al., 2024 [59]
Metal Alloys (e.g., Co-Cr)	High strength and durability Corrosion resistance	Implant-supported frameworks, dental models and frameworks, partial dentures	Implant Prosthodontic Discrepancy of Complete-Arch Co-Cr Implant Frameworks Manufactured Through Selective Laser Melting Additive Manufacturing Technology Using a Coordinate Measuring Machine	A recent study evaluated the implant prosthodontic discrepancies of Co-Cr SLM frameworks from three various providers using a completely edentulous maxillary cast with seven implant replicas. Measurements were taken on the x-, y-, and z-axis, and 3D gaps were calculated. Results indicated varying discrepancies among the providers, with SLM-3 exhibiting the smallest gaps overall. The z-axis showed significantly less distortion compared to the x- and y-axes across all groups. These findings suggest that the tested SLM additive manufacturing technologies produced clinically acceptable discrepancies, with attention to the z-axis distortion as a notable observation.	Revilla-León et al., 2019 [60]
Polyether ether ketone (PEEK)	High strength-to-weight ratio, excellent wear resistance	Temporomandibular joint (TMJ) devices, removable partial dentures	Application of 3 dimension-printed injection-molded polyether ether ketone lunate prosthesis in the treatment of stage III Kienböck’s disease: A case report	The patient had stage III Kienböck’s disease and underwent a 3D lunate reconstruction using a mirroring technique, followed by the insertion of a PEEK lunate prosthesis made through 3D printing and injection molding. After removing the diseased lunate bone, the prosthesis was placed in its original position. Post-surgery, improvements were observed in wrist pain, function, range of motion, grasp force, and clinical scores. X-ray results showed that the prosthesis fits well anatomically. This underscores the effectiveness of 3D-printed PEEK lunate prostheses in treating advanced Kienböck’s disease.	Yuan et al., 2022 [61]
Polylactic Acid (PLA)	Biodegradable, low toxicity, ease of printing	Temporary crowns and bridges, orthodontic appliances	Three-dimensional printing of temporary crowns with polylactic acid polymer using the fused deposition modeling technique: a case series	The case series study focused on using 3D printing technology to create temporary crowns for five patients requiring single full-coverage restorations. The process involved tooth preparation followed by 3D printing of temporary crowns using PLA polymer, with prior intraoral scanning and CAD/CAM software usage. The study showcased the versatility of 3D printing in prosthodontics by addressing various dental scenarios like cracked teeth, root canal treatments, and prosthetic restorations. The results indicated successful fabrication and application of temporary crowns without any failures or patient complaints during the temporary restoration period. While highlighting the efficiency, accuracy, and convenience of 3D printing, the study also noted limitations such as thermal degradation of PLA and surface roughness. It suggests the potential of 3D-printed temporary crowns in dental practice but suggests further research, especially in long-span prostheses and anterior teeth applications, to improve clinical outcomes and address existing limitations.	Kim et al., 2023 [62]
Polyvinyl Alcohol (PVA)	Water soluble, excellent adhesion to build layers	Support structures during printing, dissolvable models for casting and molding	Chairside fabrication of provisional crowns on FDM 3D-printed PVA Model	The study outlined the process of fabricating dental crowns using a combination of traditional stone-cast models and Polyvinyl alcohol (PVA) models with a 3D printer system. Acrylic resin and indirect resin composite materials were used to create crowns on both types of models. The fabrication process involved scanning the teeth, creating digital models, and then printing the PVA models with a 3D printer. The study assessed the accuracy and fit of the crowns using digital analysis techniques, showing that the crowns made on the PVA models had clinically acceptable levels of adaptation. This suggests that PVA models and 3D printing can be valuable tools for efficiently and accurately producing dental crowns.	Muta et al., 2020 [63]
Thermoplastic Elastomers	Flexibility and resilience, impact resistance	Soft denture liners, bite guards, occlusal splints	Initial forces and moments delivered by removable thermoplastic appliances during rotation of an upper central incisor	The study investigated the force delivery properties of three materials (Ideal Clear 1.0 mm, Erkodur 1.0 mm, Biolon 1.0 mm) used in dental appliances. Five appliances of each material were tested on an upper central incisor rotated in increments from +/-0.17 mm to +/-0.51 mm. Moments of rotation and forces of intrusion (Fz) were measured and examined. Results showed varying force magnitudes and moments depending on the material and degree of rotation, with some materials significantly affecting force delivery. The study highlighted the influence of material selection and rotation direction on the forces exerted during aligner rotation.	Hahn et al., 2010 [64]
Hybrid Composites	Combines properties of different materials, customizable properties	Hybrid denture bases, hybrid implant prostheses, hybrid restorative materials	Applications for Direct Composite Restorations in Orthodontics	The study utilized hybrid composites applied incrementally to correct various tooth size discrepancies and recontour teeth for aesthetic improvements. Case reports included scenarios where narrow or peg-shaped teeth were built up, cuspids were reshaped after orthodontic treatment, and lateral incisors were used to close spaces or replace missing central incisors. The results showed stable form and color of the composites, maintaining gingival health, and enhancing orthodontic outcomes. Overall, direct composite restorations were effective in optimizing the aesthetic and functional aspects of orthodontic treatments when indicated	Müssig et al., 2004 [65]

Applications of three-dimensional printing for dental prostheses and devices

3D technology enabled dentists to quickly create complicated dental designs with high quality and details, which gave them more treatment flexibility [2,21,39,66]. Here are some applications of 3D technology in dental prostheses.

Dental Implants

Dental implants are artificial tooth roots that help replace missing teeth, ensuring a good fit for each individual. Advancements in 3D-printing technology have improved the incorporation of implants [49]. An experimental study compared bone healing and implant stability among threaded implants, 3D-printed implants without spikes, and 3D-printed implants with spikes in four beagle dogs. Despite initial differences, all implants showed similar stability and bone integration after 12 weeks. In addition, histomorphometry analysis confirmed comparable outcomes for bone-to-implant contact among the three implant types [67].

Custom Trays

Custom trays are crucial for taking impressions, registering bites, and making temporary restorations. Usually, these trays were handcrafted from acrylic or silicone over stone casts of patients' teeth. 3D printing now offers a faster and more accurate method for producing custom trays, while digital oral scans can also be used to create tailored trays [68]. For example, Dawood et al. compared complete dentures (CDs) made with 3D-printed trays and the Gothic arch tracing technique using ARCUSdigma to traditional ones. The study revealed noteworthy variations in the bite force application, with 3D-printed custom trays showing a more uniform force distribution [69]. Additionally, an in-vitro study assessed the stability and retention strength of custom impression trays fabricated using conventional methods and additive technology. Findings indicated significant differences in dimensional stability and retention strength, with FDM trays showing higher retention strength [70].

Fixed Partial Dentures (FPDs)

FPDs or dental bridges are artificial tools that fill gaps left by missing teeth by connecting natural teeth or dental implants. In the past, fixed partial dentures were made using complex procedures such as creating wax patterns, casting in investment materials, and adding metal or ceramic veneers [71]. 3D printing has made the production of fixed partial dentures faster and more tailored. Scanning patients' mouths digitally can help create bridges using biocompatible materials such as metal alloys or polymers [72]. New research assessed the precision of temporary fixed partial dentures created on 3D-printed models with different printing angles. The findings indicated notable variations in fit between the angles, with the 60° angle showing the most optimal marginal fit at about 39.302 µm and the 30° angle exhibiting the best internal fit at about 72.876 µm [73].

Removable Partial Dentures (RPDs)

RPDs serve as a solution for replacing missing teeth. These dentures are artificial teeth mounted on a framework that connects to the existing teeth and gums, facilitating ease of removal and cleaning [74]. A recent systematic review showed that RPDs generally had high satisfaction rates (50-81%), influenced by factors such as age, gender, RPD experience, denture type (metal vs. flexible), and attachments. Flexible dentures were preferred, and attachments such as magnets and implants improved satisfaction. Common sources of dissatisfaction included pain, aesthetics, and cleanliness issues [75]. Conventional techniques for creating partial dentures are time-consuming and require manual processes, such as waxing and casting. Digital scans are quicker and more accurate to produce denture components straight from materials such as polymers or metals [76]. A clinical trial examined the effects of 3D-printed dentures versus plaster-cast dentures on patients with dental problems. Findings indicated that the group using 3D printing had a decreased number of tender points, improved chewing function, and increased satisfaction levels [77].

CDs

CDs replace all teeth in the upper or lower dental arch to restore oral function and aesthetics for patients who have lost all their natural teeth [78]. There is an ongoing discussion regarding the effectiveness and contentment of traditional full dentures compared to those made through 3D printing. A comparison of digital and traditional methods in creating CDs was conducted in 14 laboratory studies. Digital CDs typically exhibited acceptable adaptation and occlusal accuracy, occasionally surpassing traditionally fabricated CDs. Nevertheless, abnormalities were often observed in areas, for example, the posterior portion of the palate and borders of the oral cavity. Denture accuracy is greatly impacted by factors such as fabrication technique, the CAD-CAM system used, and long-term performance. However, there is a requirement for further clarification on the influence of factors such as casting shape, CAD-CAM parameters, and analytical techniques on the precision of digital CD. Current evidence does not definitively prove the superiority of CAD-CAM milling and 3D printing in denture accuracy [79].

Maxillofacial Prostheses

Maxillofacial prosthetics assist in restoring facial appearance and oral functions following surgery or injury. Progress in 3D printing has enhanced precision, shortened surgery time, and boosted outcomes for maxillofacial surgery patients. Recent studies show that 3D-printed models can aid in treatment planning and radiography [80].

Orthodontic Appliances

Orthodontic devices are used to fix dental irregularities such as malocclusions and misalignments. Traditional orthodontic appliances are large which makes patients uncomfortable and needs frequent adjustments. [81] Recently, 3D printing has been used to fabricate customised aligners and retainers that perfectly fit each patient. Furthermore, integrating computational intelligence and augmented reality with 3D printing techniques has enhanced the accuracy and reliability of orthodontic models, demonstrating a high level of improvement and customization between 3D-printed models and traditional plaster casts in long-term orthodontic treatment supervision [82].

Resolution and accuracy of three-dimensional printing technologies

Resolution in 3D printing is detail and precise level in an object. It is determined by feature size or layer thickness, for example, higher resolution allows for finer details, smoother surfaces, and accurate geometries. Different 3D printing modalities offer variable resolutions; photopolymerization-based ones such as SLS and DLP provide high resolution with thin layer thicknesses and produce detailed shapes. Additionally, material extrusion-based technologies such as FDM have lower resolution due to visible layer lines and rough surfaces [81,82].

Accuracy in 3D printing refers to the degree of conformity between a fabricated component and its corresponding digital design. Several factors influence accuracy, including material properties, thermal effects during the build process, and post-processing treatments. To ensure dimensional and geometric fidelity, rigorous quality control protocols are essential. This involves comparing the printed component to the CAD model using precise metrology techniques. Instruments such as coordinate measuring machines (CMMs), laser scanners, and optical microscopy are employed to assess dimensional accuracy, surface roughness, and microstructural characteristics [83-85]. 

It is crucial to consider the impact of process-induced variations on part quality. Factors such as material shrinkage, thermal expansion, and build orientation can significantly affect the mechanical properties and dimensional stability of the printed component. A comprehensive understanding of these variables is necessary to optimize the 3D-printing process and achieve the desired level of accuracy [85,86].

Discussion

Lately, 3D technology has impacted different medical fields, such as surgery, genetics, and dentistry. It aids in creating visual models of human anatomy, enhancing understanding of surgical procedures, and interpreting genomes. Moreover, it has affected the education process by promoting the innovation of visual models that improve students' remembrance of information [87].

Despite the numerous benefits of 3D printing, there are specific challenges, for example, the requirement for continuous training and education for dental professionals. Healthcare providers must be updated with the latest updates in digital workflows, and software tools. They must also follow strict rules and recommendations to ensure the safety, effectiveness, and compatibility of 3D-printed prosthetics and equipment. Quality assurance protocols, testing materials, and post-processing techniques are necessary to maintain high levels of care and keep patients satisfied [26,88].

Furthermore, while 3D printing offers distinctive flexibility, it has limitations, as mentioned before. Clinicians must select appropriate materials and technologies based on patient needs and treatment objectives [24,89].

Bioprinting and tissue engineering are regenerative dentistry by creating patient-specific dental tissues and organs that mimic native tissue properties. Progress in regenerating dental tissues using dedicated bio-inks and printing techniques with stem cells shows promise for enhancing patient outcomes and restoring damaged structures [90].

Artificial intelligence (AI) and machine learning algorithms' integration into 3D printing workflows shows remarkable potential for enhancing medical applications by analysing patient data to create tailored treatment plans, enhance implant placement based on anatomy, and predict orthodontic outcomes [91].

In research on individuals with periodontitis, they were divided into groups receiving only AI, AI combined with human counselling (AIHC), or a control group. They applied the DENTAL MONITORING tool for scanning and evaluation. AI and AIHC demonstrated superior enhancement in periodontal metrics compared to the control group, while AIHC showed better outcomes in decreasing probing depth and attachment level. It is indicated that AI-aided counselling is advantageous for periodontitis patients [92].

Future Directions and Opportunities

To progress 3D printing in prosthodontics, various suggestions can be proposed for evaluating 3D-printed prostheses. More studies are required to assess the quality, effectiveness, and durability of different 3D-printing materials, techniques, and software for prosthodontics. In terms of expenses, it is suggested to perform thorough cost-benefit evaluations to assess the economic viability of implementing 3D-printing technology in prosthodontics among various socioeconomic groups. Furthermore, we recommend educational and training programs to improve the skills of dental professionals in utilizing 3D-printing technology.

## Conclusions

This review comprehensively explored the applications of 3D-printing technology in prosthodontics, highlighting its ability to create high-resolution, complex dental prostheses. The integration of digital design and CAD/CAM modelling with 3D printing has revolutionized prosthodontic workflows, enhancing accuracy, efficiency, and treatment outcomes. Various materials now cater to diverse clinical needs, offering biocompatibility, strength, and aesthetics. While challenges such as high costs and material limitations remain, ongoing advancements in materials science and printing technology are expected to overcome these barriers. The future of 3D printing in prosthodontics is promising, with the potential to further transform the field, offering patients personalized, high-quality restorations.
